# Alienation from school and cyberbullying among Chinese middle school students: A moderated mediation model involving self-esteem and emotional intelligence

**DOI:** 10.3389/fpubh.2022.903206

**Published:** 2022-09-13

**Authors:** Xiong Gan, Pinyi Wang, Chen Huang, Hao Li, Xin Jin

**Affiliations:** ^1^Department of Psychology, College of Education and Sports Sciences, Yangtze University, Jingzhou, China; ^2^Department of Psychology, College of Education and Sports Sciences, Yangtze University College of Technology and Engineering, Jingzhou, China

**Keywords:** alienation from school, cyberbullying, self-esteem, emotional intelligence, middle school students

## Abstract

As an extension of traditional bullying behavior, cyberbullying behavior emerges with the increasing popularity of the internet, and seriously affects the health of middle school students. However, just a few studies have explored the impact of the school factor on cyberbullying and its underlying mechanisms. Therefore, the purpose of this study is to explore the potential mediator (i.e., self-esteem) and potential moderator (i.e., emotional intelligence) of the relationships between alienation from school and cyberbullying. Five hundred and seventy five Chinese middle school students participated in the study (45.74% male) and completed self-report questionnaires regarding alienation from school, cyberbullying, self-esteem, and emotional intelligence. Correlation analysis showed a positive correlation between alienation from school and cyberbullying. Mediation analysis indicated that alienation from school positively predicts individual cyberbullying, and self-esteem partly mediated the association. Meanwhile, emotional intelligence moderated the pathway from alienation from school to cyberbullying. Specifically, the effect of high alienation from school on cyberbullying was weaker for middle school students who reported high emotional intelligence. The findings of this study expose the influence of the school factor and individual factors on cyberbullying, which has potential preventive and intervention value for youth cyberbullying.

## Introduction

According to the China Internet Network Information Center's 48th Statistical Report, as of June 2021, the number of internet users in China had reached 1.011 billion, with 12.3% of internet users between the ages of 10 and 19 (1). With the rising popularity of the internet, the issue of cyberbullying among middle school students is becoming an extremely incisive social problem (2). Cyberbullying is defined as an intentional, aggressive, and repetitive behavior perpetrated by a more powerful individual against someone more vulnerable through the use of technology such as the internet, social media, and cellular phones (3). Because of the concealed characteristic of cyberbullying, in which individuals believe they can hide their identity and attack others without responsibility, it has become so common that we can't ignore it (2). Considering the psychological characteristics of adolescents, when they lack sufficient personal resources or experience to deal with various psychological stressors, they are easily influenced by the internet (3). Many studies have confirmed the negative impact of these cyberbullying behaviors on middle school students (4), especially on the victims (5, 6). Less attention is paid to the bully, the person who is the perpetrator of cyberbullying (2). While paying attention to the victims, we should also know why the bullies carry out the bullying behavior through the internet.

In recent years, researchers have begun to investigate the influence of school factors on school bullying, but the correlation between the school factor and cyberbullying is not clear (5, 7). Based on the attachment theory and ecological system theory, the current study investigated the influence of the school factor (alienation from school) and individual factors (self-esteem, emotional intelligence) on middle school students' cyberbullying, and provided scientific and effective prevention and intervention suggestion for middle school students who carry out cyberbullying, which has an important practical implication.

### Alienation from school and cyberbullying

According to the ecological system theory (8), school is the main place for middle school students to study and grow, and an important subsystem that affects their physical and mental development. As a central element of the school experience, school connectedness is related to behavioral outcomes (9, 10). School connectedness refers to how students generally feel about their school and connect positively with their peers, teachers, and other adults in the school community (11). For adolescents in school, everyone wants to build a trusting relationship with teachers and peers. However, adolescents' psychological needs are unsatisfied when they feel alienated from school (12). Brown (13) defined alienation from school as a sense of distress or loss that occurs when students feel a disconnection or distance between themselves and others. Feelings of school alienation lead students to perceive life and school as broken and incomplete and thus feel powerless to cope with the expectations placed on them by life and school. Students in school life, due to the normal relationship alienation, have a series of powerless, isolated, meaningless, self-alienation, and other negative emotions. According to attachment theory (14), when individuals feel insecure and have difficulty meeting their inner needs, they may seek other sources, such as the internet, to compensate for the emotional hunger they cannot satisfy in the real world, resulting in a corresponding increase of cyberbullying (15, 16). In addition, we can understand the relationship between alienation from school and cyberbullying behavior with social support. As a previous result, alienation from school is a significant negative predictor of social support (9), which in turn is a negative predictor of adolescent cyberbullying (17). Other researchers put forward that the sense of alienation from school makes adolescents more prone to have emotional regulation problems, which is in line with the process model of emotional regulation (4, 18). The more difficult it is for students to regulate their emotions, the more likely they are to engage in cyberbullying (19).

Previous studies on cyberbullying have focused on individual factors (3, 4), and underestimated the school factor. However, the individual characteristics of adolescents and the school environment both influence cyberbullying behavior at different levels (20). Given the above theories and related research, we think that if students feel alienation from school, they will experience a sense of isolation, pointlessness, impotence, and other negative emotions, which may increase cyberbullying behavior. Previous research had found that aloneness is a significant predictor of adolescent cyberbullying (21, 22). Other researchers have demonstrated the predictive effect of bullying on alienation without exploring the reverse path (5). Therefore, it is critical to investigate the possible mechanism of the relationship between adolescent alienation from school and cyberbullying to obtain a better understanding of the relationship between them.

### Self-esteem as a mediator

Rosenberg defined self-esteem as “a favorable or unfavorable attitude toward the self” (23). The attachment theory of self-esteem (24) holds that self-concept and self-worth develop through repeated interactions with significant others (e.g., teachers and peers). It is conducive for students to the formation of a good attachment when they feel closely connected with the school, and then the development of self-esteem. On the contrary, individuals with a strong sense of alienation from school can't get enough care and love, and form a secure attachment relationship, which leads them more likely to form low self-esteem (15). Similarly, according to Maslow's hierarchical theory of needs (25), if students' belonging needs (such as school belongingness) are not satisfied, their higher-level needs (such as self-esteem needs) will be damaged. Previous research has shown that alienation from school is negatively associated with self-esteem in adolescents (26, 27). On the other hand, the self-defense view of self-esteem diminishing (28) points out that individuals have the tendency to maintain high self-esteem, and when faced with low self-esteem caused by alienation from school, they will have the motivation to protect and defend their self-consciousness and tend to do destructive behaviors (such as bullying) (29). In addition, per the diathesis-stress model (30), adolescents with a personality vulnerability (e.g., low self-esteem) may exhibit more maladaptive behaviors (e.g., cyberbullying). Previous studies have shown that self-esteem has a significant negative predictive effect on cyberbullying (29, 31, 32), indicating that the lower the level of self-esteem, the more likely middle school students are to exhibit cyberbullying behavior.

The self-systematic belief model (33) holds that external risk factors influence the outcome of adaptation through an individual's self-systematic belief. Therefore, alienation from school may have an impact on cyberbullying through self-esteem. Few previous studies have explored the connection between these variables simultaneously, but many studies have shown that loneliness, as a component of alienation from school, affects cyberbullying through self-esteem (21, 22). This suggests that self-esteem may be a connection between alienation from school and cyberbullying.

### Emotional intelligence as a moderator

Although alienation from school may directly or indirectly affect cyberbullying through self-esteem, it does not influence adolescents equally. Mayer and Salovey (34) define emotional intelligence as the ability to monitor one's own and other's emotions, discriminate among them, and use this information to guide one's thinking and actions. Middle school students with higher emotional intelligence have a wide range of emotional regulation strategies and are less likely to seek external sources (such as the internet) to alleviate their negative emotions (4). Emotional intelligence is a significant protective factor on aggressive behaviors (18, 35). For instance, Yu (36) found that adolescents' emotional intelligence remitted the relationship between low levels of self-esteem and aggressive behavior. According to the risk and proactive factor framework (37), cyberbullying is the result of a dynamic interaction between risk and protective factors. Therefore, emotional intelligence may be the protective intrapersonal trait that enhances resiliency when adolescents are confronted with risk factors (e.g., buffers the effects of alienation from school and low self-esteem on cyberbullying). Specifically, individuals with alienation from school or low self-esteem may be protected by emotional intelligence and can effectively regulate their emotions when facing problems, and reduce cyberbullying behavior.

**Figure 1:**
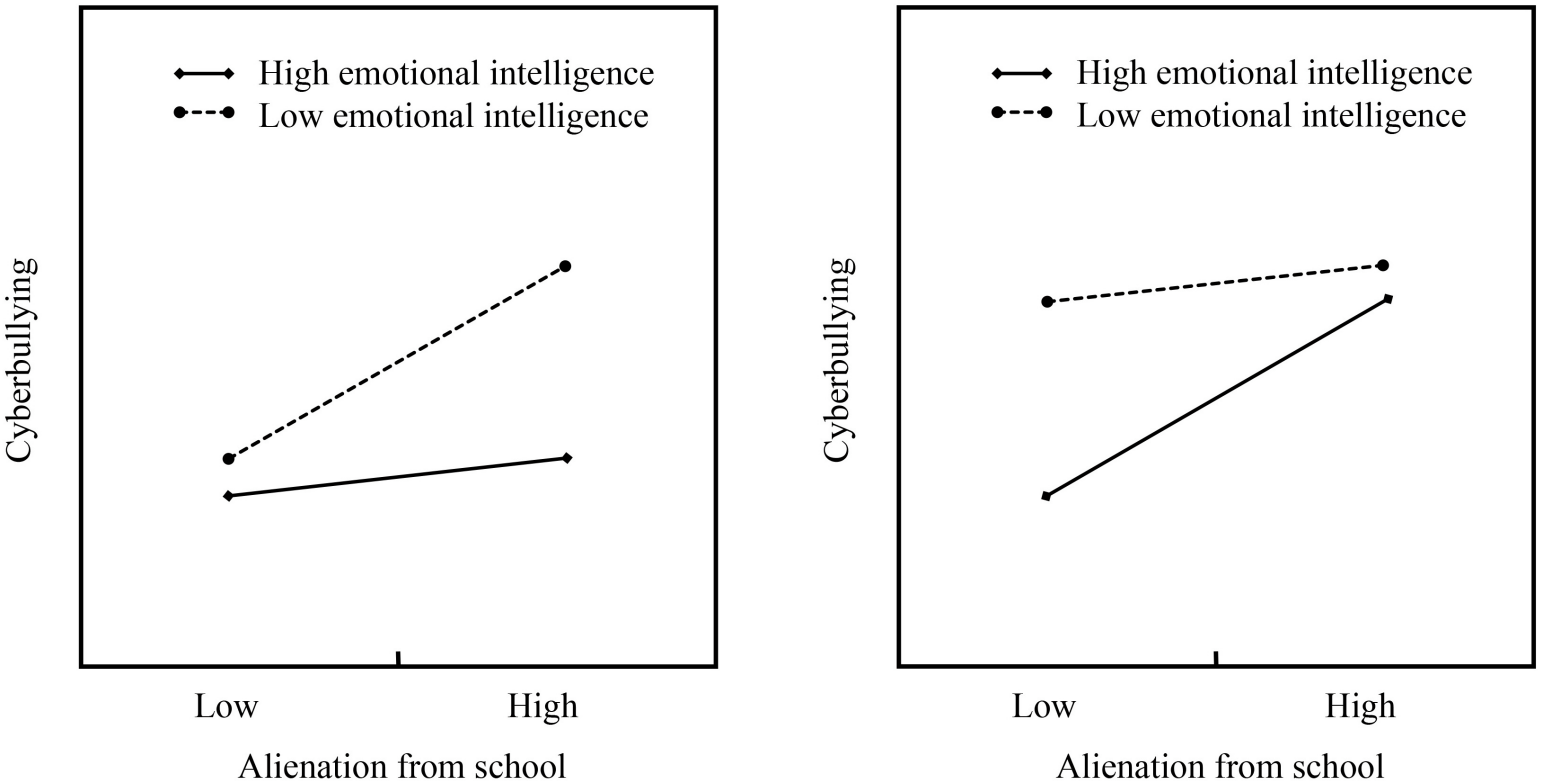
Hypothetical alienation from school × Emotional intelligence interaction impacts. **(A)** On the right is the risk-buffering model, and **(B)** on the left is the reverse risk-buffering model.

Although the above findings suggest that emotional intelligence may moderate the direct and indirect relationships between alienation from school and cyberbullying, theoretical models differ on how to explain this moderating effect. The risk-buffering model (Figure 1A) suggests that personal assets can mitigate the adverse effects of environmental risk factors on children's development. According to this model, the harmful effects of environmental risks will be weaker for individuals with higher levels of personal assets (38). On the contrary, the reverse risk-buffering model (Figure 1B) argues that once environmental risks reach a certain level, personal assets may lose their ability to offset risks (the protective effect of personal assets will be inhibited in the face of high environmental risks). In this case, for individuals with a higher level of personal assets, the adverse impact of environmental risks will be stronger. Different models of moderate indicate different practical meanings (38). The risk-buffering model suggests that intervention programs for adolescent emotional intelligence will benefit individuals who suffer from alienation from school. The reverse risk-buffering model suggests that the role of emotional intelligence should not be overstated and the adverse effects of alienation from school should not be ignored (10). Therefore, it is of great significance to distinguish the moderate pattern of emotional intelligence for better prevention and intervention of cyberbullying behavior.

To our knowledge, the moderation effect of emotional intelligence on the association between antecedent risk (e.g., alienation or low self-esteem) and adolescent cyberbullying has hardly been studied. To fill the gap, we aimed to examine whether middle school students with higher (compared to lower) emotional intelligence would be less likely to display cyberbullying in the context of alienation from school or lower self-esteem.

### The present study

**Figure 2:**
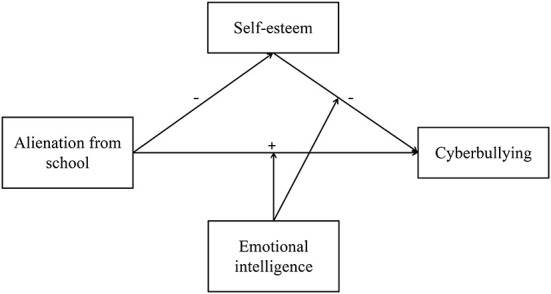
Proposed mechanism of the association between alienation from school and cyberbullying.

We aimed to examine the influence of alienation from school, self-esteem, and emotional intelligence on cyberbullying and its internal mechanism in the same model, based on the above mentioned literature review and related theories. We believe that alienation from school is a predictor of cyberbullying, meaning that it can significantly predict cyberbullying in middle school students (hypothesis 1). The connection between alienation from school and cyberbullying is mediated by self-esteem (hypothesis 2). Emotional intelligence will decrease the direct effect of alienation from school on individual cyberbullying (hypothesis 3a) as well as the second part of the mediation process namely the effect of self-esteem on individual cyberbullying (hypothesis 3b). The whole proposed model is depicted in Figure 2.

Taken together, self-esteem and emotional intelligence are expected to shed light on the relationship between alienation from school and cyberbullying. Identifying the mechanism by which alienation from school is linked to cyberbullying has crucial implications for theory and prevention, and is a step closer to developing an integrative framework to understand the complex relationships among variables in our study.

## Methods

### Participants

The current study used data from 575 (45.75 % male) middle school students from an ongoing longitudinal study. We recruited participants from two middle schools through simple random cluster sampling in Hubei Province, China. Due to the different arrangements of class activities, participants came from certain classes of grades 7 (0.2 %), 8 (45.9 %), 9(7.0 %), seniors 1 (1.0 %), and 2 (45.9 %). Reflecting the demographics of the area, 48.9% of the adolescents came from rural areas, 15.8% from county seats, and 35.3% from cities.

### Procedures

The study was approved by The Research Ethics Committee of the Psychology, College of Education and Sports Sciences, Yangtze University. A group test was conducted in the classroom, with graduate students trained in professional psychology acting as the conductor. With the informed consent of students and their parents or legal guardians involved, an anonymous questionnaire survey was conducted, and the questionnaire was collected after they finished. This study is part of an ongoing longitudinal study, this time with data collected in March 2021 and all items completed within 20 min. All participants were told that their answers will be kept confidential and they can quit at any time if they feel uncomfortable and were required to complete all items independently throughout the process.

### Measures

#### Alienation from school

The questionnaire of alienation from school compiled by Li et al. (39) was used. The questionnaire contains six items and all items were rated on a 4-point scale ranging from 1 (strongly disagree) to 4 (strongly agree). Item scores were averaged to create a composite of alienation from school, with higher scores indicating higher levels of alienation from school. Studies have shown that this questionnaire has good reliability and validity among Chinese adolescents (38). In the current study, the questionnaire's Cronbach's alphas coefficient was 0.86.

#### Cyberbullying

Cyberbullying was measured with six items complied by Lam and Li (40). All items were rated on a 7-point scale ranging from 0 (never) to 6 (more than or equal to 6 times). The average score of all items was calculated, with higher scores indicating a higher frequency of showing cyberbullying behavior. Studies have shown that this questionnaire has good reliability and validity among Chinese adolescents (6, 41). The questionnaire's Cronbach's alphas coefficient was 0.78 in this study.

#### Self-esteem

The Self-Esteem Scale (SES) complied by Rosenberg (23) was used, which contains ten items. All items were rated on a 4-point Likert-type scale ranging from 1 (very inconsistent) to 4 (very consistent). The average score of all items is calculated (after reverse-coding when necessary), with higher scores indicating higher levels of self-esteem. The Chinese version of this scale has been widely used and has demonstrated good reliability and validity (42). In the present study, Cronbach's alphas coefficient was 0.61.

#### Emotional intelligence

The questionnaire of emotional intelligence revised by Wang (43) was used, which contains 33 items. According to David (44), this study uses four dimensions of emotional intelligence: self-management of emotions, social skills, empathy, and utilization of emotions to assess the level of emotional intelligence. This questionnaire contains twelve items, and all items were rated on a 5-point Likert-type scale ranging from 1 (very inconsistent) to 5 (very consistent). Each dimension's average score is calculated, with higher scores indicating higher levels of emotional intelligence. This scale has demonstrated good reliability and validity in samples of Chinese adolescents (45). In the present study, the Cronbach's alphas coefficients for the four subscales and the overall scale were 0.72, 0.81, 0.89, 0.82, and 0.91, respectively.

### Statistical analysis

Prior research has found that adolescents' gender and grade are related to cyberbullying (2, 21, 22). Thus, we controlled for these demographic variables in our statistical analyses. For descriptive statistics and correlation analysis, SPSS26.0 was utilized. We used the SPSS macro PROCESS to examine mediation effect and moderating effect (46). We used bootstrapping with 2,000 iterations to test the statistical significance of the mediation effect. All variables were standardized before testing and dummy coding was used for all categorical variables (e.g., gender and grade). The interaction product was created by multiplying the two predictors and the moderator. When an interaction effect was significant, simple slope analyses (1 *SD* above and below the mean of the moderator) were conducted (47).

## Results

### Control and inspection of common method bias

Because the data were collected through self-reporting, there may be a common method bias. Some approaches, such as reverse scoring and anonymous filling, were used to control the process to decrease the influence of the common method bias. Harman single factor test was used to inspect this bias (48). The results reveal that the eigenvalues of seven factors are more than 1, and the variance explained by the first factor is 20.44%, which is much less than the critical value of 40%, indicating that there is no significant common method bias in this study.

### Descriptive statistics

Means, standard deviations, and correlations are displayed in Table 1. As regard to covariates, females had lower alienation from school level (*r* = −0.15, *p* < 0.01) than males. Students in higher grades had higher alienation from school level (*r* = 0.10, *p* < 0.05), lower emotional intelligence (*r* = −0.19, *p* < 0.01). Alienation from school was positively correlated with cyberbullying (*r* = 0.21, *p* < 0.01), meaning that students who experienced higher levels of alienation from school were more likely to show a higher frequency of cyberbullying behavior. Both alienation from school and cyberbullying are negatively correlated with self-esteem (*r*_1_ = −0.16, *p* < 0.01; *r*_2_ = −0.13, *p* < 0.01), indicating that students who experienced higher levels of alienation from school and showed a higher frequency of cyberbullying behavior were more likely to have low levels of self-esteem.

**Table 1:** Descriptive statistics and correlations for all variables.

**Variables**	***M***	***SD***	**1**	**2**	**3**	**4**	**5**	**6**
1. Gender	1.54	0.50	1					
2. Grade	3.94	1.94	0.09*	1				
3. Alienation from school	−0.05	0.94	−0.15***	0.10*	1			
4. Cyberbulling	−0.14	0.67	−0.06	0.02	0.21***	1		
5. Self-esteem	0.04	0.87	0.05	−0.05	−0.16***	−0.13**	1	
6. Emotional intelligence	0.03	0.99	0.01	−0.19***	−0.25***	−0.21***	0.33***	1

Gender was dummy coded such that 1 = male, 2 = female, and Grade was dummy coded such that 1 = grade 7, 2 = grade 8, 3 = grade 9, 4 = senior 1, 5 = senior 2, 6 = senior 3.

***p < 0.001, **p < 0.01, *p < 0.05.

### Mediating effects of self-esteem

**Figure 3:**
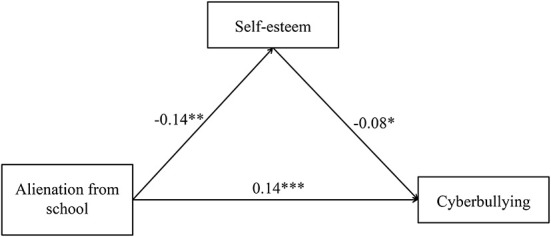
The mediating role of self-esteem. All covariates were held constant during this analysis but are not presented for reasons of simplicity. ***p < 0.001, **p < 0.01, *p < 0.05.

Model 4 of the SPSS macro program PROCESS was used to test the mediating effect of self-esteem. The results are shown in Table 2. After controlling for gender and grade, alienation from school is a significant positive predictor of cyberbullying (β = 0.15, *p* < 0.001), and hypothesis 1 is proved. When both alienation from school and self-esteem enter the regression equation, alienation from school can significantly positively predict cyberbullying (β = 0.14, *p* < 0.001), and significantly negatively predict self-esteem (β = −0.14, *p* < 0.01). Self-esteem can significantly negatively predict cyberbullying (β = −0.08, *p* < 0.05). The bias correction Bootstrap method test shows that self-esteem has a significant mediating effect between alienation from school and cyberbullying, *ab* = 0.01, *SE* = 0.01, 95% *CI* [0.01, 0.21], proving hypothesis 2. As shown in Figure 3, the mediation effect accounted for 7.47% of the total effect of alienation from school on cyberbullying.

**Table 2:** The mediating model of alienation from school and cyberbullying.

**Variables**	**Model 1**		**Model 2**		**Model 3**	
	**Cyberbullying**		**Self-esteem**		**Cyberbullying**	
	**β**	***t***	**β**	***t***	**β**	***t***
Gender	−0.05	−0.86	0.07	0.90	−0.04	−0.77
Grade	0.00	0.11	−0.02	−1.01	0.00	0.01
Alienation from school	0.15***	5.00	−0.14**	−3.62	0.14***	4.60
Self-esteem					−0.08*	−2.48
*R* ^2^	0.05		0.03		0.06	
*F*	9.40***		5.71***		8.65***	

***p < 0.001, **p < 0.01, *p < 0.05.

### Moderating effects of emotional intelligence

Model 15 of the SPSS macro program PROCESS was used to test the moderating effect of emotional intelligence. Gender and grade were controlled and all variables were standardized. The results are shown in Table 3, alienation from school significantly positively predicted cyberbullying (β = 0.10, *p* < 0.01), and the interaction term between alienation from school and emotional intelligence significantly negatively predicted cyberbullying (β = −0.17, *p* < 0.001), meaning that emotional intelligence moderated the relationship between alienation from school and cyberbullying, proving hypothesis 3a. However, the predictive effect of the interaction term between self-esteem and emotional intelligence on cyberbullying is insignificant (β = 0.01, *p* > 0.05), indicating that emotional intelligence doesn't moderate the relationship between self-esteem and cyberbullying.

**Table 3:** The moderating model of alienation from school and cyberbullying.

**Variables**	**β**	***t***	***SE***	***LLCI***	***ULCI***
Gender	−0.03	−0.53	0.05	−0.14	0.08
Grade	−0.02	−1.58	0.01	−0.05	0.01
Alienation from school	0.10**	3.21	0.03	0.04	0.15
Self-esteem	−0.03	−1.01	0.03	−0.10	0.03
Emotional intelligence	−0.12***	−3.93	0.03	−0.18	−0.06
Alienation from school * Emotional intelligence	−0.17***	−4.43	0.03	−0.17	−0.07
Self-esteem * Emotional intelligence	0.01	0.18	0.03	−0.05	0.06
*R* ^2^	0.11				
*F*	10.02***				

***p < 0.001, **p < 0.01, *p < 0.05.

**Figure 4:**
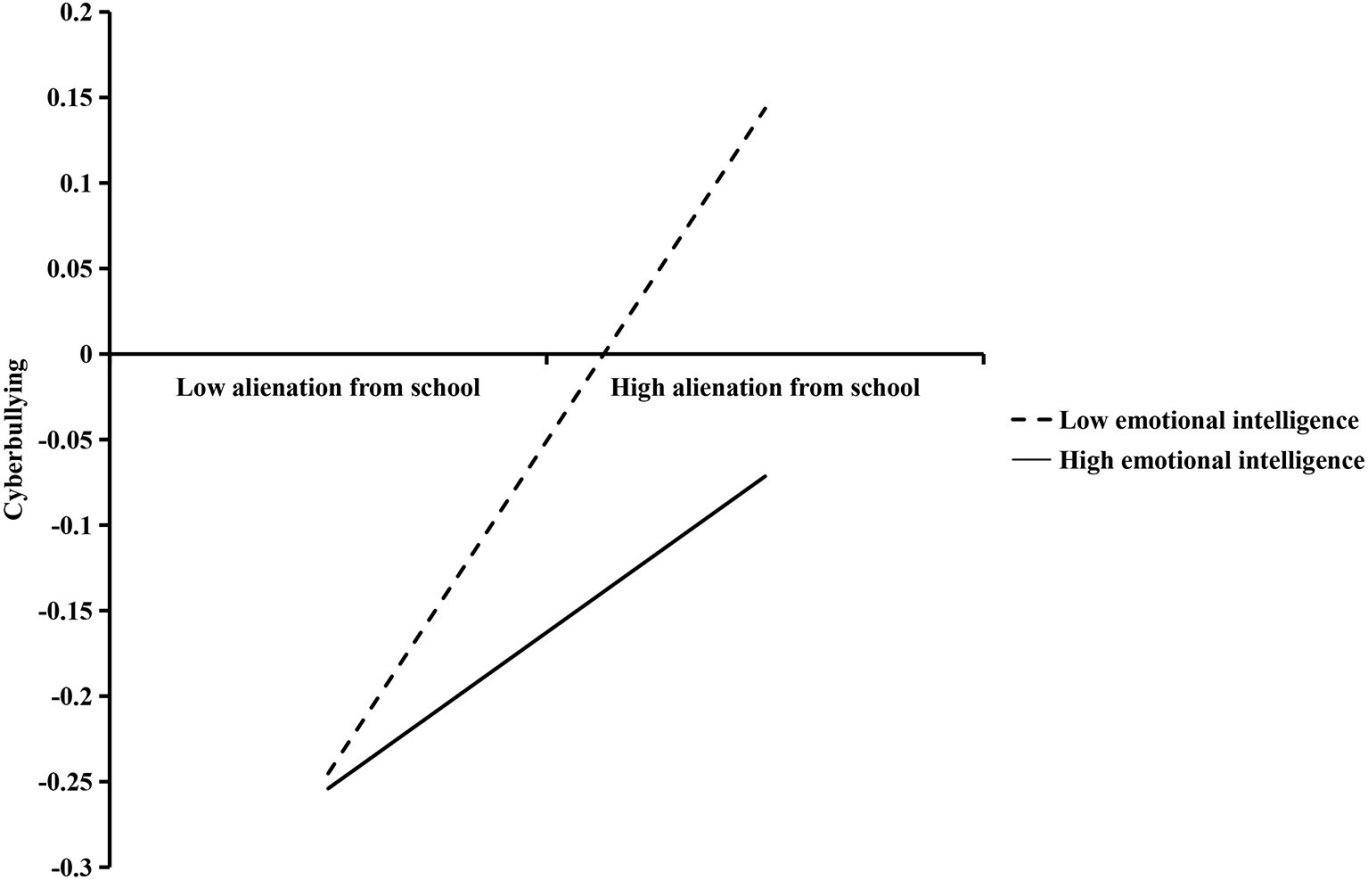
The moderating role of emotional intelligence in the effect of alienation from school on cyberbullying.

Follow-up analyses (presented in Figure 4) tested simple slopes of alienation from school at lower (1 *SD* below mean) and higher (1 *SD* above mean) levels of emotional intelligence to understand the nature of the interaction. For students with a low level (−1 *SD*) of emotional intelligence, alienation from school was associated with higher cyberbullying. For students with a high level (+1 *SD*) of emotional intelligence, the relationship between alienation from school with cyberbullying was relatively weaker. Thus, high emotional intelligence seemed as a protective factor while low emotional intelligence was a risk factor in the association between alienation from school and cyberbullying.

## Discussion

The current researcher focused on the impact of the school factor and individual factors on cyberbullying in middle school students. This study not only explores the influence of the school factor on cyberbullying but also considers the mechanism of individual factors.

### Alienation from school and cyberbullying

The findings demonstrate that the higher the level of alienation from school, the higher the adolescents' cyberbullying level, which is in accordance with related findings (5) and also makes extension to it. A large number of studies have confirmed that alienation from school has many negative influences on students (12, 49), such as dropping out and being isolated. When individuals experience a high level of alienation from school, they will feel lonely and insecure, and then seek the internet to meet their needs. Students are easily influenced by the internet and show cyberbullying behavior. These finding is accordance with attachment theory and lay the foundation for more research into the mediating roles of self-esteem and moderating roles of emotional intelligence in the connection between alienation from school and cyberbullying.

### Self-esteem as a mediator

Our findings suggest that alienation from school has a negative predictive effect on self-esteem, which in turn increases the likelihood of cyberbullying among middle school students. This finding suggests that self-esteem may be an important explanation mechanism for the link between alienation from school and cyberbullying. The mediating effect of self-esteem is consistent with related research (21, 22). Alienation from school can predict self-esteem (50, 51), and self-esteem can predict cyberbullying in a significant way (32, 52). According to sociometer theory (53), self-esteem is an internal monitor of how much a person is valued by others. If students lack a strong relationship with their teachers or classmates, they may view themselves negatively and thus show a lower level of self-esteem. This finding is also consistent with Maslow's hierarchy theory of needs (25), that is, if an individual's belongingness needs are not satisfied, his self-esteem needs will also be violated. Previous research has shown that alienation from school is negatively associated with self-esteem in adolescents (26, 27). In the current study, it is manifested as the negative predictive effect of alienation from school on self-esteem. This finding has particular significance in the context of Chinese culture. China is a collectivist society that places great emphasis on interpersonal relationships (54), so alienation from school may be more harmful. On the other hand, self-esteem plays a key role in the process of adapting to the external environment. Poor self-perception makes students less confident, and they tend to search themselves on the internet to vent their negative feelings in reality (15). This finding suggests that self-esteem may be an important explanatory mechanism for the link between alienation from school and cyberbullying. However, it is worth noting that self-esteem plays a partial mediating role in the relationship between alienation from school and cyberbullying, indicating that the influence of alienation from school on cyberbullying may be mediated by other factors, and extensive research is needed.

### Emotional intelligence as a moderator

In addition to the indirect path, our results also partially supported hypothesis 3. As expected, emotional intelligence moderated the pathway from alienation from school to cyberbullying. To be specific, students with higher emotional intelligence were less likely to be cyberbullied than those with lower emotional intelligence among students when experiencing high alienation from school. In other words, higher emotional intelligence as a protective factor eased the negative impact of alienation from school on cyberbullying, which fits the risk-buffering model (38). Previous studies have shown that emotional intelligence moderates the relationship between risk factors and developmental outcomes (55–57). Therefore, we believe that emotional intelligence can make individuals rationally deal with the sense of meaninglessness and loneliness caused by alienation from school, and promote students to adopt positive strategies, thus contributing to the reduction of internet uncivilized behaviors (e.g., cyberbullying). However, emotional intelligence did not moderate the relationship between self-esteem and cyberbullying. One possible explanation is that improvements in emotional intelligence don't effectively satisfy self-improvements. Thus, although emotional intelligence is a protective factor, it does not mitigate the adverse effects of low self-esteem. This finding adds to evidence that low self-esteem is a significant risk factor for cyberbullying, and emotional intelligence is not immune to the risks posed by low self-esteem. More research is needed in the future to examine other protective factors that may buffer the relationship between low self-esteem and cyberbullying, such as peer relationships (58).

From a practical standpoint, these findings highlight the importance of clarifying such processes and the protective role of emotional intelligence to improve prevention and intervention that can alter at various stages.

### Contributions and limitations

There are some important contributions of this study. First, this study explores the influence of the school factor and individual factors on cyberbullying, as well as the underlying mechanism. Therefore, it is particularly important to establish the school connection for adolescents. For example, creating a good school climate, optimizing the teacher-student relationship, strengthening the home-school connection, training students' anti-frustration quality, etc., can be used to improve students' sense of alienation from school. Second, the mediating effect of self-esteem suggests that intervention programs can focus on restoring self-esteem damaged by alienation from school and further improving mental health levels. Furthermore, the stress-buffering model of emotional intelligence suggests that the improvement of emotional intelligence will help students who suffer from alienation from school to reduce cyberbullying behavior.

Despite the above-mentioned contributions, some limitations of this study should be mentioned. First, this study uses a cross-sectional design to explore the causal relationship between variables, and the causality relationships can't be delineated. A longitudinal design is expected to be used in the future to better explore the causal relationship between variables. Second, we used the self-reporting method to collect data, which may be affected by social desirability. Future research should use multiple methods and multiple informants to increase the validity of the findings. Third, this study found that the relationship between alienation from school and cyberbullying was partially mediated, suggesting that a further understanding of this relationship requires information on other mediators. Fourth, research has identified the complex nature of the bullying experience, in that some bullies are also victims (59). Whether cyberbullying shares this characteristic and the mechanisms underlying such group behavior is also something we need to explore in the future. Finally, the current study was conducted with a sample of Chinese middle school students, and future research should include cross-cultural samples of adolescents to test the generalizability of our findings.

## Conclusion

This study explores the mediating role of self-esteem and moderating role of emotional intelligence in alienation from school and cyberbullying, and draws the following conclusions: Alienation from school could positively predict cyberbullying; The relationship between alienation from school and cyberbullying is mediated by self-esteem; The relationship between alienation from school and cyberbullying is moderated by emotional intelligence.

## Data availability statement

The original contributions presented in the study are included in the article/Supplementary material, further inquiries can be directed to the corresponding authors.

## Ethics statement

The studies involving human participants were reviewed and approved by the Research Ethics Committee of the Psychology, College of Education and Sports Sciences, Yangtze University. Written informed consent to participate in this study was provided by the participants' legal guardian/next of kin.

## Author contributions

XG and XJ designed the study. XG contributor in accessing fund support. CH collected the data. XG, PW, and HL analyzed and interpreted the data and jointly wrote the manuscript. All authors read and approved the final manuscript.

## Funding

This work was supported by Youth project of Science and Technology Research Plan of Department of Education of Hubei Province in 2020 (Q20201306), Project of Social Science Foundation of Young Scholar Support Plan of Yangtze University in 2020 (2020skq24), and Project of Social Science Foundation of Yangtze University in 2021 (2021csy15).

## Conflict of interest

The authors declare that the research was conducted in the absence of any commercial or financial relationships that could be construed as a potential conflict of interest.

## Publisher's note

All claims expressed in this article are solely those of the authors and do not necessarily represent those of their affiliated organizations, or those of the publisher, the editors and the reviewers. Any product that may be evaluated in this article, or claim that may be made by its manufacturer, is not guaranteed or endorsed by the publisher.
